# Repurposing FDA-approved drugs as multi-target neuroprotective agents for Alzheimer’s disease via computational screening and experimental validation

**DOI:** 10.1038/s41598-026-46708-2

**Published:** 2026-04-07

**Authors:** Khemjira Phemphunananchai, Pornthip Waiwut, Jutarop Phetcharaburanin, Pattaporn Poonsawas, Chantana Boonyarat

**Affiliations:** 1 Faculty of Pharmaceutical Sciences, Khon Kaen University, 123, Mittraphap Road, Nai-Muang, Muang District, Khon Kaen, 40002 Thailand; 2 Faculty of Pharmaceutical Sciences, Ubon Ratchathani University, 85, Sathollamark Road, Mueang Si Kai, Warin Chamrap District, Ubon Ratchathani, 34190 Thailand; 3 Department of Systems Biosciences and Computational Medicine, Faculty of Medicine, Khon Kaen University, 123, Mittraphap Road, Nai-Muang, Muang District, Khon Kaen, 40002 Thailand

**Keywords:** Alzheimer’s disease, Drug repurposing, Virtual screening, BACE1 inhibitors, Multi-target therapy, Biochemistry, Drug discovery, Neuroscience

## Abstract

β-site APP-cleaving enzyme 1 (BACE1, a β-secretase) is a key aspartyl protease that initiates the proteolytic processing of the amyloid precursor protein (APP) to form the amyloid-β (Aβ) peptide. Given that Aβ aggregation and plaque formation are a central pathological feature of Alzheimer’s disease (AD), BACE1 remains a critical therapeutic target. Furthermore, the complexity of AD pathology necessitates the identification of novel multi-target agents. This study employed a structure-based virtual screening, targeting the BACE1 approach to identify the potential BACE1 inhibitors from FDA-approved drug scaffolds derived from the ZINC database. Top-ranked candidates were subsequently validated through extensive 500 ns molecular dynamics (MD) simulations and *in vitro* assays of BACE1 inhibition activity. Furthermore, multi-AD-related target profiling of candidates was conducted using molecular docking and a series of *in vitro* assays. Among them, ZINC000019796155 emerged as a promising multi-target compound. Biochemical analysis revealed that ZINC000019796155 exhibited moderate inhibitory effects against BACE1. Subsequent assays confirmed its capacity to inhibit butyrylcholinesterase (BuChE) action and Aβ aggregation, function as a free radical scavenger, and provide neuroprotection against H₂O₂-induced oxidative stress in cellular models. Furthermore, western blot analysis elucidated the mechanism of neuroprotection, indicating that ZINC000019796155 inhibits the apoptotic pathway while simultaneously suppressing the formation of Aβ plaques and neurofibrillary tangles (NFTs). These findings highlight ZINC000019796155 as a validated scaffold with a known safety profile for AD. While further structural optimization and preclinical in vivo studies are necessary, this study underscores the potential of ZINC000019796155 as a multi-target neuroprotective agent for AD treatment.

**Supplementary Information:**

The online version contains supplementary material available at 10.1038/s41598-026-46708-2.

## Introduction

Dementia currently affects more than 55 million individuals worldwide, with Alzheimer’s disease (AD) being the predominant cause, accounting for 60–70% of cases. AD is characterized clinically by the accumulation of specific proteins in brain tissue, including amyloid β (Aβ) plaques and neurofibrillary tangles (NFTs)^1–3^ The global burden of dementia is expected to rise sharply, with more than 10 million new cases reported annually^4^ and projections estimating that the number of individuals affected will reach 74.7 million by 2030 and 152.8 million by 2050, driven largely by aging populations^5^. AD predominantly impacts individuals over 60 years of age, with prevalence increasing exponentially with advancing age.

Despite extensive research, no current therapies cure AD or completely arrest its progression. The FDA-approved medications include cholinesterase inhibitors, namely donepezil, galantamine, and rivastigmine; the N-methyl-D-aspartate (NMDA) receptor antagonist, memantine; and anti-amyloid monoclonal antibodies such as lecanemab and donanemab^6^. Cholinesterase inhibitors and memantine work by regulating neurotransmitters crucial to memory and cognitive function; however, these drugs only provide symptomatic relief. On the other hand, anti-amyloid monoclonal antibodies can slow the progression of AD but are linked to significant side effects and high costs. Moreover, neurodegeneration persists irrespective of current interventions. The consistent failure of single-target agents in advanced clinical trials has prompted a paradigm shift in AD drug design toward multi-target directed ligands (MTDLs), which address multiple interconnected pathological pathways simultaneously^7^.

A key therapeutic target in AD drug discovery remains the inhibition of Aβ aggregation and plaque formation^8^. Aβ peptides are produced by proteolytic cleavage of the amyloid precursor protein (APP) by β-site APP-cleaving enzyme 1 (BACE1), a rate-limiting aspartyl protease in Aβ plaque formation. Under AD-related conditions, overactivity of BACE1 leads to extracellular aggregation of Aβ, contributing to neurotoxicity^7^. Inhibiting BACE1 reduces Aβ plaque formation, potentially slowing AD progression and preventing neurodegeneration. Consequently, BACE1 was selected as the primary molecular target for hit compound identification in this study.

Virtual screening (VS) has become an invaluable computational approach in modern drug discovery, enabling rapid identification of compounds with favorable pharmacokinetic properties and likely blood-brain barrier permeability. In structure-based virtual screening (SBVS), libraries of compounds are docked in silico against a target’s active site to predict binding affinities and select promising candidates for experimental validation^9–11^. Several FDA-approved drugs have been identified through the VS process, including captopril, saquinavir, ritonavir, indinavir, tirofiban, dorzolamide, zanamivir, and boceprevir^10^. Therefore, virtual screening is considered a promising technique for discovering potential anti-AD agents.

With advances in bioinformatics, several databases have been developed to collect biological information from various perspectives. These databases play a significant role in drug discovery, particularly in applications such as drug repurposing^12^. An especially promising approach is in silico drug repurposing, which utilizes a database of FDA-approved drug structures to explore their potential for treating diseases beyond their original indications. The main advantage of this approach is the availability of existing preclinical data, including pharmacokinetic profiles and safety information. As a result, this strategy offers a cost-effective and time-efficient alternative in drug development, helping to accelerate progression into clinical trials^13,14^. Notably, approximately 30% of drugs approved in the US in recent years originated from repurposing efforts, including amantadine, repurposed from an antiviral to Parkinson’s disease treatment, and lithium, traditionally used for bipolar disorder.

In this study, we aimed to investigate the multi-target agent for AD treatment by employing a sophisticated SBVS pipeline to screen a library of FDA-approved drugs from the ZINC database against the active site of BACE1. Given that BACE1 is the rate-limiting step in Aβ plaque formation, it was selected as the primary filter to ensure all hits possess hallmark-related target activity before being assessed for other AD-related pathology activity. Our objective was fivefold: (1) computationally identify potential hits with high affinity for BACE1 via structure-based virtual screening and binding mode analysis, and subsequently validate complex stability using MD simulations; (2) further evaluate top candidates for a multi-target profile via preliminary molecular docking against acetylcholinesterase (AChE), butyrylcholinesterase (BuChE), and Aβ peptide; (3) experimentally validate the inhibitory activity of top candidates against BACE1; (4) assess activity against other AD-relevant targets, including AChE inhibition, BuChE inhibition, Aβ aggregation, ABTS ( 2,2′-azinobis-(3-ethylbenzothiazoline-6-sulfonic acid) radical scavenging, and neuroprotective effects; and (5) characterize the underlying neuroprotective mechanisms of the most promising hit compound through cellular signaling pathway analysis. This approach leverages the speed and efficiency of computational screening, combined with the low-risk profile of drug repurposing, to identify novel, clinically viable anti-Alzheimer’s drug candidates.

## Result

### Structure-based virtual screening for BACE1 inhibitors

**Fig. 1:**
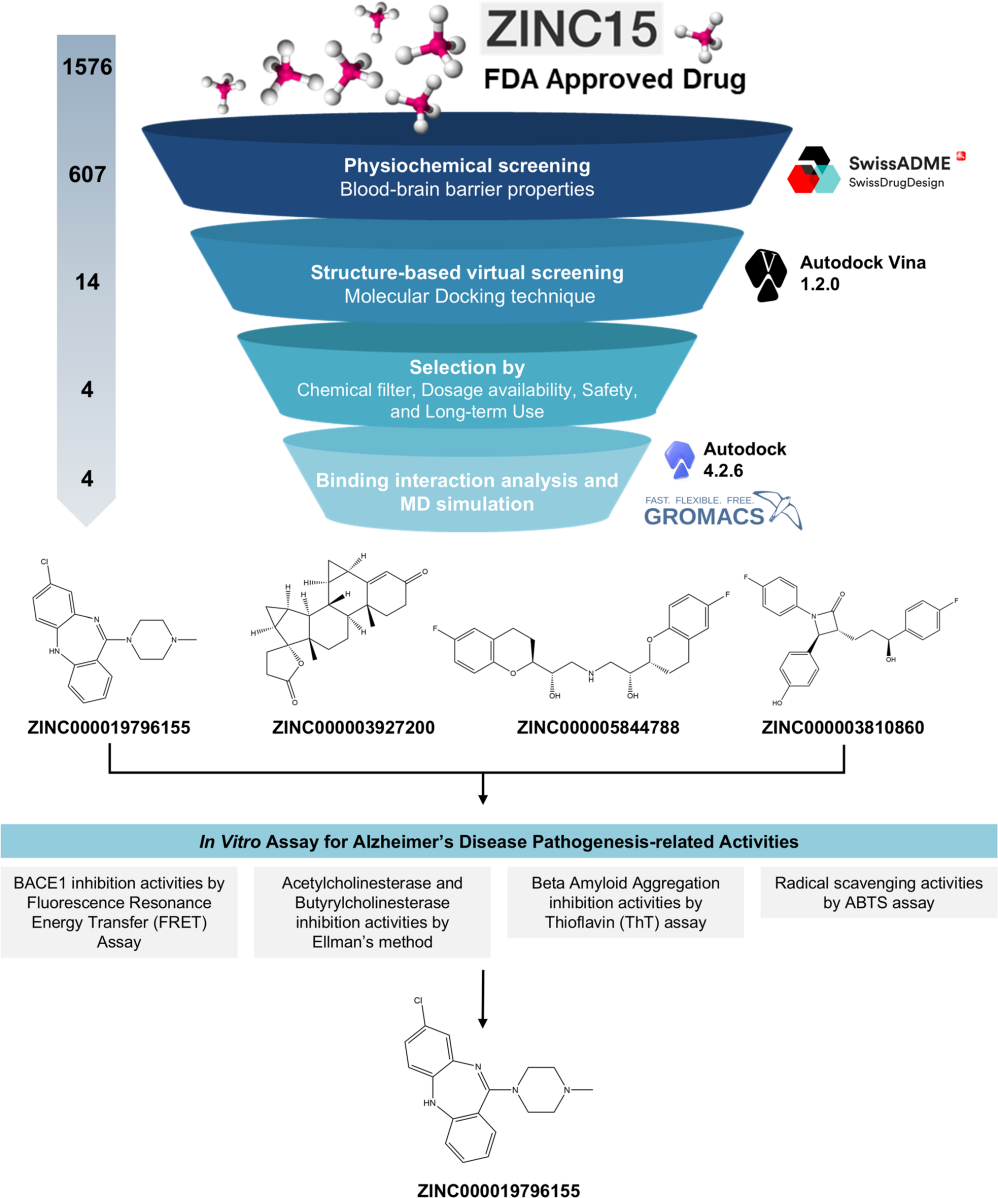
A strategy diagram for identifying the potential hit compounds for AD.

A structure-based virtual screening workflow was employed to identify potential BACE1 inhibitors from an FDA-approved drug library. As illustrated in Figs. 1 and 1,576 FDA-approved drugs in SMILES format were obtained from the Zinc15 database. To ensure central nervous system (CNS) accessibility, all compounds were pre-screened for blood–brain barrier (BBB) permeability using the SwissADME platform, yielding 607 candidates predicted to effectively cross the BBB barrier—an essential property for anti-Alzheimer’s drug candidates.

For the molecular docking process, the BACE1 crystal structure (PDB ID: 1W51) was retrieved and validated via redocking with its co-crystallized ligand, yielding a root-mean-square deviation (RMSD) of 1.34 Å. This low RMSD confirmed the reliability of the docking parameters and accuracy of the binding site configuration. Subsequent molecular docking of the 607 BBB-permeable compounds against BACE1 was performed using AutoDock Vina. Compounds were ranked by binding affinity, and those with lower binding energies than the reference BACE1 inhibitors were shortlisted, yielding 14 promising hits (Table 1).

**Table 1:** List and binding affinity of hit compounds and known inhibitors obtained from structure-based virtual screening.

Molecule	Binding affinity (kcal/mol)	Molecule	Binding affinity (kcal/mol)
ZINC000253630390	-9.9	ZINC000000601229	-9.0
ChEMBL2347204^a^	-9.8	ZINC000000538658	-8.9
ChEMBL3695737^a^	-9.8	ZINC000003797541	-8.9
ZINC000003927200	-9.7	ZINC000003810860	-8.8
ZINC000000537928	-9.4	ZINC000000004893	-8.7
ZINC000036701290	-9.4	ZINC000019796155	-8.7
ZINC000052716421	-9.4	ZINC000100015048	-8.7
ZINC000005844788	-9.2	ChEMBL2177912^a^	-8.6
ZINC000001542199	-9.1	ChEMBL3688641^a^	-8.6

^a^ positive standard (known BACE1 inhibitors).

Among these, four compounds, including ZINC000019796155, ZINC000003927200, ZINC000005844788, and ZINC000003810860, were selected for further evaluation based on favorable docking scores, chemical properties, availability of oral dosage form, and documented clinical long-term safety (see Supplementary Table S1). These compounds have been previously approved for long-term therapeutic use, suggesting their potential suitability for repurposing in chronic neurodegenerative conditions such as Alzheimer’s disease^15–18^. The selected compounds were further investigated for the positioning and binding interaction in BACE1’s active site. Ultimately, the compounds with the favorable active-site positioning and binding interaction were chosen for MD simulations to validate molecular dynamics stability.

### Molecular interactions analysis of top ligands on BACE1

Molecular interactions analysis revealed that all four selected compounds, ZINC000019796155, ZINC000003927200, ZINC000005844788, and ZINC000003810860, showed promising binding affinities with a binding energy of -8.59, -9.53, -10.66, and − 8.38, respectively. While BACE1 inhibitor IV, the positive standard for the in vitro BACE1 inhibition assay, exhibits a binding energy of -10.48 kcal/mol, as shown in Table 2.

In addition to the binding score, the binding positioning and interaction were used to screen the potential of the promising compounds. The binding interaction analysis indicated that all four selected compounds successfully occupied the active site of BACE1 and formed multiple hydrogen bonds and hydrophobic bonds with several residues in the active site’s sub-pocket, as shown in Fig. 3a-e. In the BACE1 active site, catalytic aspartic acids (Asp32/Asp228) are the crucial residues responsible for the APP proteolytic processing. ZINC000019796155 showed the pi-anion hydrophobic interaction with Asp228, while ZINC000005844788 also forms hydrogen bonds with Asp 32. These results demonstrated the potential BACE1 inhibition activity of ZINC000019796155 and ZINC000005844788 via direct catalytic dyad interaction. Furthermore, all of the selected compounds were positioned over the catalytic aspartic acids (as shown in Supplementary Figure S4), and were able to effectively interact with surrounding active-site residues, particularly the flap region (residues 69–74), which are crucial for controlling the accessibility of APP into the BACE1 active site^19,20^. This result suggested their inhibitory effect through direct catalytic interference and/or steric occlusion, which physically restricts the accessibility and proteolytic processing of APP substrate in the BACE1’s active site^21^.

These results revealed the potential of selected compounds through high binding affinity and favorable binding orientations in the BACE1 active site, prioritizing these compounds as the promising BACE1 inhibitors. Subsequently, the MD simulations of these hit compounds were conducted to validate the complex’ stability.

**Table 2:** Binding affinity and interactions of the top ligands and the reference inhibitor (BACE1 inhibitor IV) with the BACE1 active site (PDB ID: 1W51).

Molecule	Binding affinity (kcal/mol)	Electrostatic interactions		Hydrophobic interactions	
		Interacting residues	No. of interaction	Interacting residues	No. of interaction
ZINC000019796155	-8.59	Gln73, Gly230, Thr231	3	Leu30, Phe108, Asp228, Tyr71, Ile118	5
ZINC000003927200	-9.53	*ND*	ND	Leu30, Ile110, Tyr71, Trp115, Ile118	7
ZINC000005844788	-10.66	Asp32, Gly34, Ser35, Thr72, Gln73, Lys224, Asp228, Gly230, Thr329	8	Thr72, Phe108 Ile110, Trp115,	4
ZINC000003810860	-8.38	Gln73, Ser229, Gly230, Thr231, Thr232	7	Lys107, Ile110	4
BACE1 inhibitor IV	-10.48	Asp32, Thr72, Gln73, Gly230, Thr232	8	Leu30	1

ND: Not Detected.

### MD simulations of the BACE1-top ligand complex

**Fig. 2:**
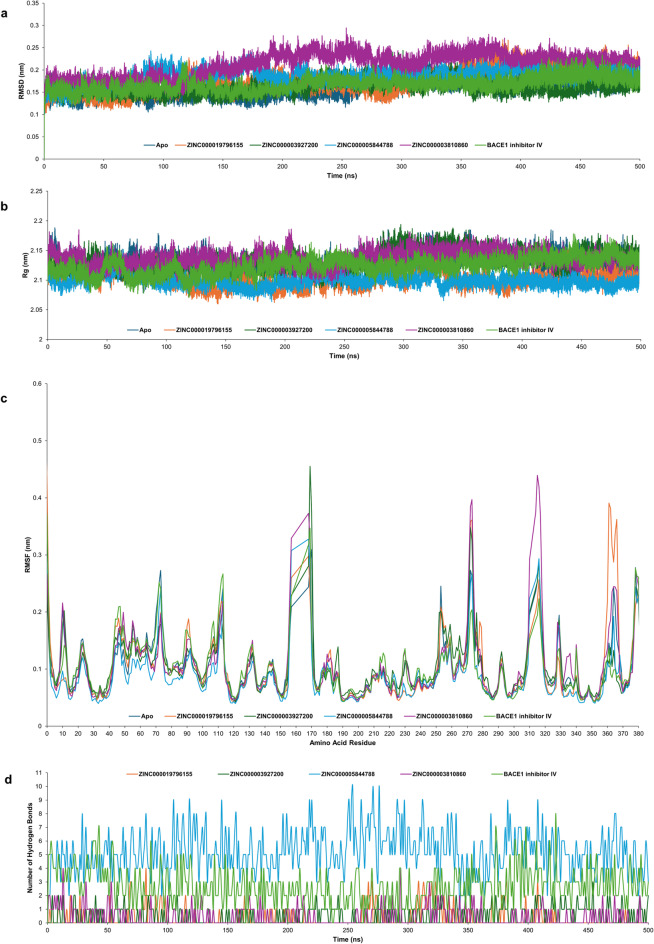
Molecular dynamics simulation analysis of the apo BACE1 structure (dark blue) and BACE1-ligand complexes, including ZINC000019796155 (orange), ZINC000003927200 (dark green), ZINC000005844788 (cyan), ZINC000003810860 (violet), and BACE1 inhibitor IV (light green). (**a**) Root Mean Square Deviation (RMSD), (**b**) Radius of Gyration (Rg), (**c**) Root Mean Square Fluctuation (RMSF), and (**d**) the number of intermolecular hydrogen bonds.

MD simulations were performed to investigate complex stability and binding behavior during a 500 ns simulations time of the top ligand-BACE1 complexes compared to the BACE1 inhibitor IV and the apo structure. The trajectories were analyzed to evaluate Root Mean Square Deviation (RMSD), Radius of Gyration (Rg), Root Mean Square Fluctuation (RMSF), and hydrogen bond, as presented in Fig. 2.

The RMSD analysis confirmed the stability of the apo and ligand-protein systems throughout the 500 ns simulation (Fig. 2a). All systems reached equilibrium, with RMSD values ranging from 0.11 to 0.29 nm. The average RMSD values of the apo form and complexed with ZINC000019796155, ZINC000003927200, ZINC000005844788, ZINC000003810860, and BACE1 inhibitor IV were 0.16 ± 0.02, 0.18 ± 0.03, 0.16 ± 0.02, 0.19 ± 0.02, 0.21 ± 0.03, and 0.17 ± 0.02 nm, respectively.

The Rg analysis was performed to determine the compactness of the BACE1 structure (Fig. 2b). The average Rg values of the apo, ZINC000019796155, ZINC000003927200, ZINC000005844788, ZINC000003810860, and BACE1 inhibitor IV were 2.13 ± 0.01, 2.10 ± 0.01, 2.13 ± 0.02, 2.10 ± 0.01, 2.14 ± 0.01, and 2.12 ± 0.01 nm, respectively. Notably, the system containing ZINC000019796155, ZINC000005844788, and the BACE1 inhibitor IV exhibited a lower Rg value than the apo structure system.

The RMSF analysis was performed to evaluate the fluctuations of amino acids in BACE1 upon ligand binding, as shown in Fig. 2c. All systems showed similar RMSF profiles, implying that there are no major structural conformation changes of the protein’s core. At the catalytic region, specifically the residues Asp32 and Asp228, all systems exhibited low RMSF values (< 0.1 nm), confirming the structural integrity of the active site. In the flap region (residues 67–75), the ligand-protein systems showed lower RMSF values than the apo form, as observed with the BACE1 inhibitor IV. Furthermore, the system contained ZINC000019796155 and ZINC000005844788 and exhibited low RMSF in the 10s loop region, comparable to that of the BACE1 inhibitor IV.

To investigate the intermolecular hydrogen bond between BACE1 and the studied ligands, hydrogen bond analysis was performed over the 500 ns trajectory, as shown in Fig. 2d. The result revealed the consistent intermolecular hydrogen formation of ligands.

According to the 500 ns MD simulations analysis, the results highlighted the robust structural stability and exceptional binding dynamics of the selected top ligands in the BACE1 active site. Based on these insights, further *in vitro* evaluations were warranted for potential candidates to validate their potential as novel therapeutics for Alzheimer’s disease.

### Preliminary screening for the potential of top ligands against AChE, BuChE, and Aβ_1−42_ using the molecular docking technique

Given the complexity of AD pathogenesis, the potential of selected top ligands against possible AD-related targets was primarily evaluated using molecular docking. The results demonstrated superior binding affinity to reference standards, as shown in Table 3.

**Fig. 3:**
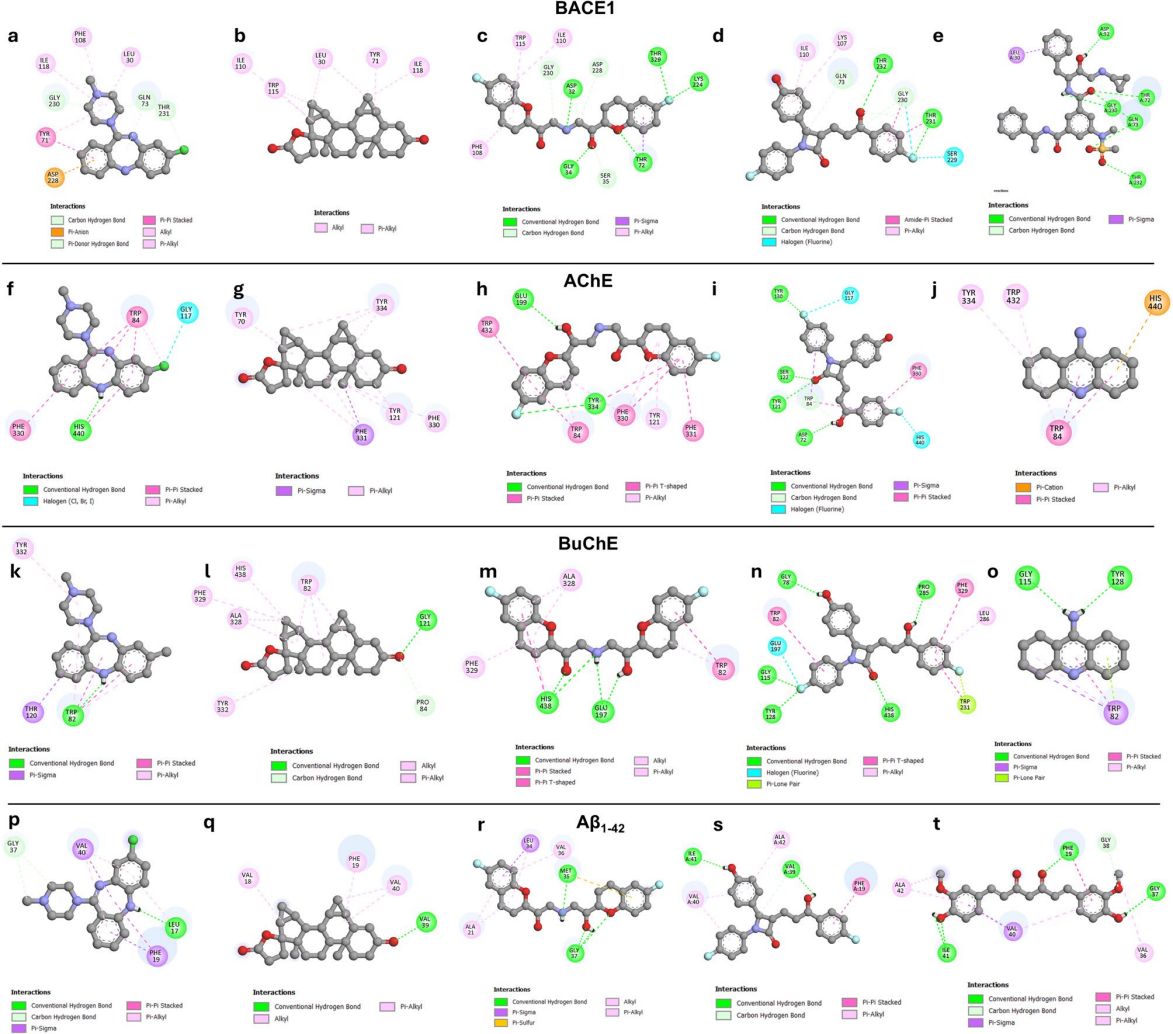
2D interaction diagrams illustrating the binding modes of topligands and reference standards against specific targets. Panels (a–e) depict interactions with BACE1; (f–j) with AChE; (k–o) with BuChE; and (p-t) with Aβ_1−42_ peptide. The ligands include ZINC000019796155, ZINC000003927200, ZINC000005844788, ZINC000003810860, respectively. BACE1 inhibitor IV is the reference standard for BACE1. Tacrine is the reference standard for AChE and BuChE. Curcumin is the reference standard for Aβ_1−42_.

For AChE, all top ligands exhibited binding affinity, ranging from − 9.72 to -13.06 kcal/mol. The binding interaction analysis showed that all top ligands and tacrine occupied the crucial active site pocket of AChE (Fig. 3f-j). Especially, ZINC000019796155 and ZINC000003810860 form intermolecular interactions with His440, one of the residues in the crucial catalytic triad. Furthermore, the binding affinity of top ligands on BuChE ranged from − 8.67 to -10.95 kcal/mol, with good occupancy in the BuChE active site (Fig. 3k-o). ZINC000003927200, ZINC000005844788, and ZINC000003810860 form an interaction with His438, a part of the catalytic residue in BuChE^22^.

For the Aβ_1−42_ peptide, ZINC000019796155, ZINC000003927200, and ZINC000003810860 showed superior binding affinity to curcumin, the reference standard, with a range from − 6.11 to -7.27 kcal/mol. All ligands bind to amino acids at residues 15–21, a crucial region for Aβ aggregation (Fig. 3p-t). ZINC000019796155 and ZINC000005844788 bound Gly37, a crucial residue in Aβ aggregation^23,24^. While ZINC000003810860 was found to bind with Ala42, a residue found to bind with Arg5 contributed to Aβ dimerization^25^. These binding interactions are consistent with curcumin’s binding mode.

These results demonstrated favorable binding affinities and interactions of the top ligands with AChE, BuChE, and the Aβ_1−42_ peptide, suggesting their potential against these specific targets. Consequently, further *in vitro* evaluations were warranted to validate these computational predictions.

**Table 3:** Binding affinity (kcal/mol) of top ligands and reference standards (tacrine and curcumin) on AChE (PDB ID:2CEK), BuChE (PDB ID: 1P0I), and Aβ_1−42_ peptide (PDB ID: 2BEG).

Index	Binding affinity (kcal/mol)		
	AchE	BuChE	Aβ peptide
ZINC000019796155	-12.25	-8.67	-6.11
ZINC000003927200	-9.72	-10.95	-7.27
ZINC000005844788	-13.06	-9.43	-3.94
ZINC000003810860	-12.01	-8.90	-6.40
Tacrine	-8.62	-6.64	ND
Curcumin	ND	ND	-6.08

ND: Not Determined.

### *In vitro* validation of BACE1 inhibition

To verify the computational predictions, the four selected compounds were subjected to an *in vitro* BACE1 enzymatic assay. All compounds demonstrated measurable BACE1 inhibitory activity, confirming their potential as BACE1 modulators. Among them, ZINC000019796155 exhibited the highest activity, with 56.74 ± 1.82% inhibition at 100 µM. This was followed by ZINC000003927200, ZINC000005844788, and ZINC000003810860 (Table 4). The experimental inhibitory potencies correlated well with their predicted binding energies. These findings confirm the reliability of the virtual screening protocol and suggest that these repurposed scaffolds, particularly ZINC000019796155, exhibited promising BACE1 inhibition.

**Table 4:** *In vitro* AD-related pathological activities of ZINC000019796155, ZINC000003927200, ZINC000005844788, and ZINC000003810860. *Data are represented as mean ± SD (n* = 3).

Index	%Inhibition at 100 µM				
	BACE1 assay	AChE assay	BuChE assay	Aβ aggregation assay	ABTS assay
ZINC000019796155	56.74 ± 1.82	31.81 ± 4.55	95.26 ± 1.09	38.41 ± 0.32	94.19 ± 0.65
ZINC000003927200	47.19 ± 1.08	22.72 ± 4.55	78.36 ± 2.02	9.95 ± 7.80	ND
ZINC000005844788	17.77 ± 4.37	36.36 ± 12.03	67.46 ± 2.34	42.04 ± 6.23	ND
ZINC000003810860	31.54 ± 2.88	33.33 ± 2.62	31.28 ± 2.70	10.63 ± 2.52	5.85 ± 3.22
BACE1 inhibitor IV^a^	56.02 ± 6.45	ND	ND	ND	ND
Tacrine^b^	ND	49.08 ± 1.97	58.48 ± 0.29	ND	ND
Curcumin^c^	ND	ND	ND	49.54 ± 4.07	ND
Trolox^d^	ND	ND	ND	ND	63.52 ± 1.48

^a^ Positive control of BACE1 assay: BACE1 inhibitor IV at 0.008 µM, ^b^ Positive control of AChE and BuChE assay: Tacrine at 0.2 µM and 0.01 µM, respectively, ^c^ Positive control of Aβ aggregation assay: Curcumin at 10 µM, ^d^ Positive control of ABTS assay: Trolox at 50 µM, ND: Not Determined.

### *In vitro* evaluation of additional Alzheimer’s disease-related activities

To further explore the multitarget potential of the identified compounds, additional *in vitro* assays related to key AD pathological mechanisms were conducted. The four compounds—ZINC000019796155, ZINC000003927200, ZINC000005844788, and ZINC000003810860—were evaluated for their ability to inhibit Aβ aggregation, scavenge free radicals, and inhibit cholinesterase enzymes (AChE and BuChE), as summarized in Table 4.

All tested compounds showed moderate AChE inhibition, with inhibitory percentages ranging from 22.72% to 36.36%. In contrast, ZINC000019796155, ZINC000003927200, and ZINC000005844788 displayed selective BuChE inhibition, exhibiting % BuChE inhibition values of 95.26 ± 1.09%, 78.36 ± 2.02%, and 67.46 ± 2.34%, respectively. The anti-Aβ aggregation assay revealed that ZINC000019796155 and ZINC000005844788 showed moderate activity, with percentage inhibition values of 38.41 ± 0.32% and 42.04 ± 6.23%, respectively. In the antioxidant assay, only ZINC000019796155 showed significant ABTS radical scavenging activity, with an inhibition of 94.19 ± 0.65%. Given its multitarget activity across key AD-related pathways, ZINC000019796155 was selected for further in-depth investigation, including the determination of IC50 values for each *in vitro* assay (Table 5).

**Table 5:** The IC_50_ values of in vitro AD-related pathological activities of ZINC000019796155. Data are represented as mean ± SD (*n* = 3).

Index	IC_50_ values (µM)				
	BACE1 assay	AChE assay	BuChE assay	Aβ_1−42_ aggregation assay	ABTS assay
ZINC000019796155	93.22 ± 7.08	> 1000	4.27 ± 1.18	0.55 ± 0.02^a^	35.22 ± 1.59
BACE1 inhibitor IV^b^	0.0076 ± 0.0003	ND	ND	ND	ND
Tacrine^c^	ND	0.17 ± 0.02	0.009 ± 0.008	ND	ND
Curcumin^d^	ND	ND	ND	0.009 ± 0.002	ND
Trolox^f^	ND	ND	ND	ND	47.59 ± 1.81

^a^ report in mM unit, ^b^ Positive control of BACE1 assay: BACE1 inhibitor IV, ^c^ Positive control of AChE and BuChE assay: Tacrine, ^d^ Positive control of Aβ aggregation assay: Curcumin, ^f^ Positive control of ABTS assay: Trolox, ND: Not Determined.

### Neuroprotective activities against oxidative stress-induced cell damage

**Fig. 4:**
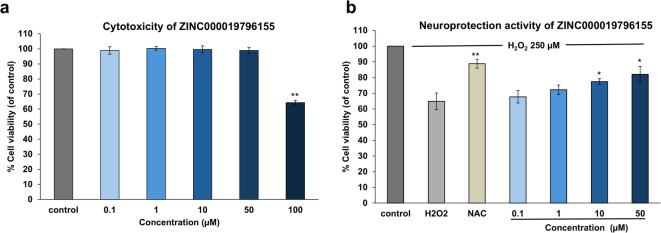
Cytotoxicity (**A**) and neuroprotection activity against hydrogen peroxide (H₂O₂)-induced cell damage (**B**) of ZINC000019796155. NAC was used as a standard. Data are means ± SD (*n* = 3). Statistical significance (*p < 0.05, **p < 0.01) indicates a significant difference compared to the control group in the cytotoxicity assay or the H₂O₂-treated control in the neuroprotection assay.

To establish appropriate concentrations for neuroprotection assays, the cytotoxicity of ZINC000019796155 was evaluated in SH-SY5Y cells. As depicted in Fig. 4A, significant cytotoxicity was observed at 100 µM, leading to the selection of 0.1–50 µM for further experiments. Subsequently, the half-maximal inhibitory concentration (IC₅₀) of H₂O₂-induced cytotoxicity was determined to be 250.3 ± 5.76 µM after a 2-hour exposure. Hence, H₂O₂ at 250 µM was used to induce oxidative stress. Pretreatment of SH-SY5Y cells with ZINC000019796155 at concentrations of 10 and 50 µM for 2 h significantly reduced cell death (Fig. 4B). The reference compounds N-acetylcysteine (NAC) also demonstrated neuroprotective activity at 100 µM under the same conditions.

### Effect of test compound on AD-related and Apoptosis-related protein expression in the SH-SY5Y neuroblastoma cell lines

**Fig. 5:**
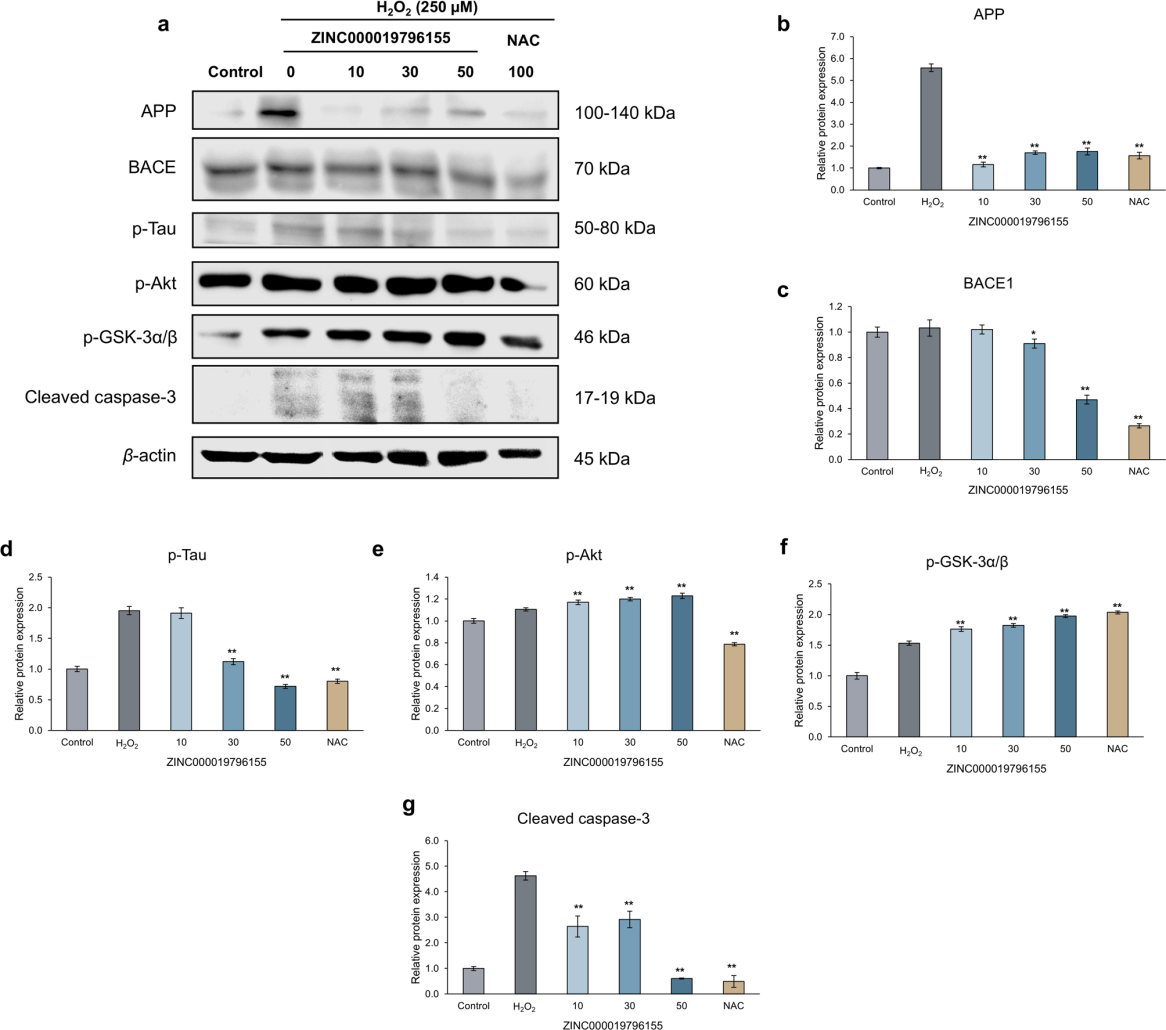
Relative protein expression of AD-related proteins in the whole SH-SY5Y neuroblastoma cell lysates under oxidative stress conditions induced by incubating with 250 µM H_2_O_2_, with and without pretreatment with ZINC000019796155 at 10, 30, and 50 µM and NAC at 100 µM as a standard. β-actin expression was used as a loading control. Data is shown as mean ± SD (*n* = 3). * *p* < 0.05 and ** *p* < 0.01 indicate the significant difference compared to H_2_O_2_ group.

To investigate the neuroprotective mechanism of ZINC000019796155 against oxidative stress-induced cell damage. We performed Western blot analysis of whole SH-SY5Y cell lysates to assess the effect of the test compound on key proteins involved in neuronal cell death and AD-related pathology, as shown in Fig. 5A.

First, the neuroprotective effect of ZINC000019796155 against oxidative stress-induced cell death was investigated. The neuroblastoma cell lines under H_2_O_2_-induced oxidative stress conditions showed increased cleaved caspase-3 expression, an apoptosis-related protein. The results indicated that pretreatment with ZINC000019796155 significantly decreased cleaved caspase-3 expression (Fig. 5G). Hence, the neuroprotective activity of ZINC000019796155 may be associated with the inhibition of the apoptosis pathway.

Furthermore, the potential of ZINC000019796155 on the AD-related protein was investigated. The expression of proteins involved in Aβ plaque and Tau formation, including amyloid precursor protein (APP), β-site amyloid precursor protein cleaving enzyme 1 (BACE), and p-Tau, was found to be increased under oxidative stress conditions. The group pretreated with ZINC000019796155 showed a significant downregulation of APP (Fig. 5B), BACE (Fig. 5C), and p-Tau (Fig. 5D), and a significant upregulation of p-Akt (Fig. 5E) and p-GSK-3alpha/beta (Fig. 5F).

According to the results, ZINC000019796155 exhibited neuroprotective activity by inhibiting the apoptotic pathway and modulating the AD-related pathological pathway, thereby inhibiting the formation of Aβ plaques and neurofibrillary tangles (NFTs). These findings revealed the potential of ZINC000019796155 as a promising anti-AD agent targeting key proteins involved in the AD-related pathogenic process.

## Discussion

In this study, we employed a structure-based virtual screening (VS) approach targeting BACE1, followed by multi-target profiling against AChE, BuChE, Aβ aggregation, and antioxidant to identify FDA-approved drugs with potential multi-target anti-AD activities, initially prioritizing inhibition of BACE1, which plays a crucial role in the proteolytic processing of the amyloid precursor protein (APP), a key event in Aβ aggregation and plaque formation, and downstream neurodegeneration involving tau pathology and oxidative damage.

The initial VS campaign utilized a curated dataset of FDA-approved drugs from the ZINC database. To prioritize compounds that cross the blood-brain barrier (BBB), a prerequisite for anti-AD therapeutics, we implemented a BBB permeability filter in SwissADME based on the BOILED-Egg model^26^. This filter identified 607 compounds predicted to cross the BBB, which were then subjected to molecular docking against BACE1. Molecular docking against the validated BACE1 was performed using AutoDock Vina 1.2.0, a robust and widely used docking tool ^27^. Validation of the docking protocol via re-docking the co-crystallized ligand yielded an RMSD of 1.34 Å, confirming the reliability of the approach^28^. The validated BACE1 structure was then used to screen 607 compounds with BBB permeability. The top 14 compounds, exhibiting the most favorable predicted binding energies compared to known BACE1 inhibitors, were selected as potential hits based on their structure-affinity relationships. Ultimately, the top-scoring compounds, four candidates (ZINC000019796155, ZINC000003927200, ZINC000005844788, and ZINC000003810860) were prioritized based on an assessment of their chemical structures, dosage availability, and documented safety profiles conducive to long-term use, warranting their safety and administration compliance in elderly populations.

Molecular interaction analysis further revealed that these hit compounds interact with the active-site residues of BACE1, which are crucial for APP cleavage and subsequent Aβ plaque formation. The interactive profile of selected compounds demonstrated their potential to interfere with the proteolytic process of the APP peptide through direct binding and/or steric occlusion. Notably, ZINC000019796155 demonstrated critical interactions consistent with known pharmacophoric features of potent BACE1 inhibitors^29^. These overall results supported all top ligands as promising BACE1 binders.

The MD simulations throughout 500 ns highlighted the exceptional binding stability of the four top ligands within the BACE1 structure. All systems rapidly reached equilibrium and exhibited stable RMSD trajectories within the acceptable threshold (< 0.3 nm), suggesting that the ligands were securely bound within the binding pocket without significant structural distortion or enzyme unfolding. The Rg profiles of the BACE1-ligand system demonstrated lower or comparable to the apo structure, indicating a compactness of the protein. In particular, the system contained ZINC000019796155 and ZINC000005844788, which exhibited significantly reduced Rg and slightly increased RMSD, consistent with an induced-fit mechanism, potentially undergoing a conformational change to optimize ligand interactions^30^. RMSF analysis highlighted that a notable low-residue fluctuation was observed in the key regions in BACE1’s active site, particularly the catalytic aspartic dyads, the flap region, and the 10s loop, indicating that the binding of top ligands rigidly anchored the crucial residues and stabilized the closed conformation of the flap region, which is crucial for substrate accessibility and enzymatic activity. Furthermore, all top ligands maintained hydrogen bonds throughout the simulation, contributing to the significant binding affinity and preventing ligand dissociation.

 The *in vitro *BACE1 inhibition assays corroborated the docking results and MD simulation. All tested compounds exhibited moderate inhibitory activity, validating the efficacy of the virtual screening approach. Among the tested candidates, ZINC000019796155 displayed the highest activity, and demonstrated clearly predicted interactions with the BACE1 catalytic site by forming the pin-anion interaction with Asp228, one of the crucial catalytic aspartic acids, and projected a piperazinyl ring to form alkyl interactions with Leu30 and Phe108, which are residues in the S1 sub-pocket, consistent with the reported key pharmacophore feature of potent BACE1 inhibitors^29^, subsequently supported by in vitro inhibitory activity.

Beyond BACE1 inhibition, the identified compounds exhibited multitarget activities relevant to AD pathology. Moderate AChE inhibition, alongside potent BuChE inhibition, was observed, predominantly in ZINC000019796155, ZINC000003927200, and ZINC000005844788. Although all the top ligands exhibited promising binding modes with crucial residues in both cholinesterases, in *vitro* results demonstrated high selectivity for BuChE over AChE. A previous study of the active-site gorge dimensions found that the active site of BuChE (501.91 Å^3^) is significantly larger than that of AChE (302.31 Å^3^)^31^. The surface volume of the top ligands was predicted to range from 292.71 to 371.20 Å^3^ (Supplementary Table S2). These compounds are likely to fit into the larger cavity of BuChE than into the smaller cavity of AChE. However, this dual cholinesterase inhibition profile may provide symptomatic relief by enhancing cholinergic neurotransmission, complementing the disease-modifying potential of BACE1 inhibition. Furthermore, moderated anti-Aβ_1−42_ aggregation and significant antioxidant activities were also observed, especially by ZINC00001979615. This compound demonstrated a binding affinity of -6.11 kcal/mol to Aβ_1−42_, comparable to the reference standard. The binding mode analysis revealed the Pi-Pi stacking interactions^32^ with Phe19, a residue in the central hydrophobic cluster in Aβ_1−42_, which is the crucial region contributing to Aβ aggregation^33^, explaining the moderate inhibitory activity. ZINC00001979615 also exhibited a potent antioxidant activity, consistent with the existence of a secondary amine within the tricyclic system. The adjacent aromatic ring provided a conjugated system to stabilize the nitrogen radical, facilitating the hydrogen-atom transfer (HAT) mechanism. Ultimately, these results highlighted the capability of ZINC00001979615 to intervene at multiple pathological nodes of AD.

In the Aβ hypothesis, APP is primarily cleaved by β-secretase and γ-secretase, resulting in the release of Aβ^34^. Aβ is released into the extracellular space and subsequently aggregated into oligomers, β-pleated sheets of fibril and plaque, which are insoluble and toxic to neurons^7,35,36^. Aβ plaques have been identified as the primary hallmark of AD associated with neurofibrillary tangle (NFTs) formation, and oxidative stress, resulting in neuronal death^37^. The result revealed that ZINC000019796155 showed potential to Aβ aggregation and plaque formation by inhibiting BACE1 activity and Aβ aggregation.

According to the cholinergic hypothesis, AChE and BuChE are pivotal in regulating acetylcholine (ACh) levels, a key neurotransmitter involved in cognitive function. In a healthy brain, AChE is the primary enzyme for ACh hydrolysis and is highly expressed in the hippocampus and the neocortical area of cerebal cortex^38^. In contrast, BuChE is expressed at lower levels than AChE in the hippocampus and temporal cortex^39^. In the AD brain, the formation of Aβ plaques significantly causes cholinergic neuron death, leading to a significant decline in ACh levels, which is associated with cognitive decline in AD^40^. Changes in AChE and BuChE activity have been observed in the AD brain. As AD pathology progresses, AChE activity remains stable or decreases, whereas BuChE activity gradually increases^41,42^. Therefore, BuChE was assumed to have a dominant role as the compensatory enzyme for ACh hydrolysis in moderate-to-severe AD. The result demonstrates that ZINC000019796155 exhibits selective inhibition of BuChE, which may offer therapeutic benefits by providing symptomatic relief in moderate-to-severe AD. Additionally, BuChE’s higher selectivity may help avoid peripheral cholinergic side effects often associated with strong AChE inhibition.

In the Oxidative stress hypothesis, Aβ plaques have been found to induce the generation of reactive oxygen species (ROS). The hydroxyl radical (HO•) generated from H_2_O_2_ is the most reactive one in the ROS family^43^. These radicals cause lipid peroxidation, resulting in two lipid peroxidation products, including mitochondrial toxins hydroxynonenal (HNE) and malondialdehyde, which damage crucial mitochondrial processes. These sequences result in inducing neuronal apoptosis^7^. Hence, a compound with radical-scavenging activity is a promising agent for reducing radical-mediated damage and neuronal death. The results revealed that ZINC000019796155 exhibited radical scavenging activity. Furthermore, the neuroprotection activity assay against H_2_O_2_-induced oxidative stress cell damage demonstrated that ZINC000019796155 exhibits neuroprotective activity, partially due to its antioxidant properties. Nevertheless, additional pathways might also contribute to this neuroprotective effect.

The underlying mechanism of neuroprotective activity was elucidated by using Western blot analysis. Our findings revealed that cleaved caspase-3 was significantly downregulated in the cell group pretreated with ZINC000019796155. Previous research found that cleaved caspase-3 expression was elevated in the AD brain and co-accumulated with Aβ plaques and NFTs. These findings indicated that cleaved caspase-3 was identified as the crucial hallmark of apoptosis in AD^44,45^. Furthermore, the H_2_O_2_-induced oxidative stress in SH-SY5Y cells was found to cause cell death through the mitochondrial apoptotic pathway. In this pathway, the excessive ROS could cause mitochondrial membrane disruption, resulting in the release of cytochrome C and activation of apoptotic proteins, including caspase-3^46^. The result revealed that the H_2_O_2_-treated group showed upregulation of cleaved caspase-3, consistent with previous research. As a result, our findings indicated that ZINC000019796155 possessed neuroprotective effects through radical scavenging and anti-apoptotic activity.

Furthermore, this study investigated the effect of ZINC000019796155 on the expression of AD-related proteins involved in Aβ plaque formation (APP and BACE), and NFTs formation (p-Tau, p-Akt, and p-GSK-3alpha/beta) using Western blot analysis. The formation of Aβ plaque and NFTs led to the release of ROS via NMDA receptors. In addition, ROS was also found to elevate the formation of Aβ plaque and tau phosphorylation^45^. Our results showed that H_2_O_2_-treated neurons exhibited upregulation of APP, BACE, and p-Tau, indicating increased Aβ plaque formation and Tau phosphorylation. The pretreatment of ZINC000019796155 significantly downregulated the expression of APP, the precursor protein of Aβ, and BACE, a crucial enzyme in Aβ production. These findings suggest that ZINC000019796155 may possess neuroprotective effects by inhibiting Aβ plaque formation.

For NFTs formation, Neurofibrillary tangles (NFTs) are the intracellular accumulation of hyperphosphorylated tau. The phosphorylation of tau was regulated by various kinases and phosphatases. Many kinases, including glycogen synthase kinase-3b (GSK3β) and cyclin-dependent kinase-5 (CDK5), have been implicated in the abnormal phosphorylation of tau in AD^47^. Especially, GSK3β has been reported to increase tau phosphorylation in transgenic mice^48^. In the resting state, this kinase was found to be active in a dephosphorylated form, and the inactivation of GSK3 is regulated by phosphorylation at serine21/9 from the upstream kinases, including Akt^49^. Our results revealed that pretreatment with ZINC000019796155 significantly upregulated p-Akt and p-GSK-3alpha/beta, leading to downregulation of p-Tau. These findings suggested that ZINC000019796155 possessed neuroprotective effects and decreased NFTs by inhibiting GSK3β and reducing the level of phosphorylated tau.

In summary, this study integrated structure-based virtual screening against BACE1 with *in vitro* AD-related activity assays and Western blot analysis to identify ZINC000019796155 as a promising multitarget-directed ligand for AD. The identification of this FDA-approved backbone provided a significant advantage regarding safety and bioavailability for further investigation. While the current IC_50_ of BACE1 inhibition activity (~ 93 µM) indicates moderate potency compared to the nanomolar reference standard, this scaffold may offer a strategic advantage. Several clinical trials of potent BACE1 inhibitors have been terminated due to safety concerns, suggesting that the complete inhibition of BACE1 activity is associated with adverse events, including cognitive worsening^50^. Conversely, the partial BACE1 knock-out mice (BACE1^+/−^·5XFAD) study demonstrated that a partial reduction of BACE1 activity significantly reduces Aβ aggregation without impairment of long-term potentiation or synaptic plasticity^51^. Therefore, the moderate inhibition profile of ZINC000019796155 may provide a safer therapeutic window, avoiding adverse effects, while achieving efficacy for AD treatment^52^. Furthermore, the multi-AD-target activity of this compound offers broader therapeutic benefit against complex AD pathology. Although the proximity dose between the *in vitro* activities and cytotoxicity in SH-SY5Y cells (approximately 100 µM) suggests a narrow therapeutic index. Given that ZINC000019796155 is an FDA-approved drug with an established human safety profile^53,54^. This finding suggests a dosage boundary rather than immediate exclusion. Consequently, this scaffold serves as a validated starting point that may require structural optimization to improve efficacy, holding significant potential for translation into clinical trials. Further investigation should prioritize optimized leads to widen the therapeutic index, followed by efficacy and safety profiling in *in vivo *models, paving the way for its ultimate assessment in clinical trials. Overall, this work underscores the power of computational and structural biology approaches to accelerate the identification of potential drug candidates for complex diseases such as Alzheimer’s disease.

## Conclusion

This study successfully integrated *in silico* strategies and *in vitro* biochemical evaluations to identify ZINC000019796155 as a promising multi-target-directed ligand for Alzheimer’s disease. The compound exhibited moderate *in vitro* BACE1 inhibitory activity, potentially offering a safer therapeutic window compared to high-potency inhibitors, alongside the ability to inhibit BuChE, reduce Aβ_1−42_ aggregation, and scavenge free radicals, highlighting its potential to address multiple facets of AD pathology. Furthermore, ZINC000019796155 modulates key AD-related proteins in the Aβ plaque and neurofibrillary tangle (NFT) formation. While the therapeutic index suggests the dosage boundary, the FDA-approved status of this compound provides a significant potential for further structural optimization to improve potency and selectivity. These findings provide a strong rationale for further structural optimization, followed by preclinical investigation in relevant animal models, paving the way for potential clinical translation. Ultimately, this work underscores the synergistic power of computational and structural biology methodologies in expediting the discovery of repurposed drug candidates for complex neurodegenerative disorders like Alzheimer’s disease.

## Materials and methods

### Material

The test compounds were purchased from Pacific Science (Pacific Science Co., LTD., Thailand). Aβ_1−42_ and fetal bovine serum (FBS) were acquired from Gibthai (Gibthai Co., Ltd., Thailand). BACE1 activity detection kit (CS0010), β-secretase inhibitor IV, tacrine, acetylthiocholine iodide (ATCI), butyrylthiocholine iodide (BTCI), trolox, DMEM/F12, 5,5’-ditthiobis (2-nitrobenzoic acid) (DTNB), and 2,2′-azinobis-(3-ethylbenzothiazoline-6-sulfonic acid) (ABTS) were obtained from Sigma-Aldrich (SM Chemical Supplies Co., Ltd., Bangkok, Thailand). Antibodies were purchased from Cell Signaling and Santa Cruz.

### Virtual screening

#### Dataset preparation

A dataset of 1576 FDA-approved drug structures in SMILES file format was obtained from the Zinc15 database^55^. Physicochemical property predictions were performed using SWISSADME (http://www.swissadme.ch/) and admetSAR (http://lmmd.ecust.edu.cn/admetsar2/). The molecular volume and surface area were calculated by using Biovia Discovery Studio 2025. Compounds predicted to penetrate the blood-brain barrier (BBB) were selected for further investigation.

#### Molecular target model preparation

The crystal protein structure of BACE1 (pdb code: 1W51)^56^ was chosen based on its established utility in identifying BACE1 inhibitors in previous in silico studies^57–59^. This crystal protein structure was obtained from the RCSB Protein Data Bank (https://www.rcsb.org/). Preparation and validation of the protein-ligand docking model were conducted using AutoDock 4.2.6^60^. All small molecules and water molecules in the structure were removed, and polar hydrogen atoms were added. For docking calculation, Gasteiger charges were added. The grid map was set up with a grid box size of 76 × 70 × 66 Å and a unit grid spacing of 0.375 Å. The center of the grid box was located at 66.951, 45.226, and 7.286. Model validation was performed using a self-docking approach, in which the native ligand was redocked to its original binding site. A root-mean-square deviation (RMSD) below 2.0 Å was considered acceptable, indicating reliable docking accuracy.

#### Virtual screening and molecular docking

After screening for BBB permeability, the 3D structures of the screened compounds were obtained from the Zinc database. These structures were further investigated for binding affinity using molecular docking with the AutoDock Vina (version 1.1.2)^20^. Docking was performed in the command prompt, and the folder containing the Receptor.pdbqt, Ligand.pdbqt, conf_vs.txt (grid data), Vina_windows.pl, Ligand.txt, vina_split.exe, and vina.exe files was navigated to. Then, the following command was used.

> perl vina_vs_win.pl.

Ligand file: Ligand.txt.

The results were reported as binding affinity (kcal/mol) in the log.txt file. The binding affinities of known BACE1 inhibitors were used as cutoff values, including ChEMBL2347204, ChEMBL3695737, ChEMBL2177912, and ChEMBL3688641.

The top-scoring compounds were further screened for their geriatric patient safety and clinical compliance in Alzheimer’s disease patients. The three strict criteria were used to prioritize compounds, including physicochemical Compliance (Lipinski’s Rule of 5), oral dosage availability, and safety for oral chronic use.

#### Binding interaction analysis using molecular docking

Following the screening, the selected compounds were visually screened based on their capabilities to interact with the catalytic dyad and/or other crucial residues in the BACE1 active site. The 3D structures of selected top ligands were docked into the prepared BACE1 (PDB ID: 1W51) by using the AutoDock 4.2.6 program^60^. The grid map was set up with a grid box size of 76 × 70 × 66 Å and a unit grid spacing of 0.375 Å. The center of the grid box was located at 66.951, 45.226, 7.286. The interaction with the catalytic dyad (Asp32 and/or Asp228) was prioritized as the primary criterion for compound selection. For the top-scoring compound that did not form a direct interaction with the catalytic dyad, the positioning of the compound in BACE1’s active site was evaluated as the secondary criterion. The compound, effectively occupied in the active site and positioned over the catalytic dyad, was also included as a potential compound.

For AChE, the crystal structure of AChE (PDB ID: 2CEK)^61^ was prepared by removing small molecules and water molecules and incorporating hydrogen atoms, using AutoDock 4.2.6. The grid map was set up with a grid box size of 66 × 66 × 70 and a unit grid spacing of 0.375 Å. The center of the grid box was located at 4.211, 65.875, and 66.579. For BuChE, the crystal structure of BuChE (PDB ID: 1P0I)^62^ was used. The grid map was set up with a grid box size of 58 × 60 × 56 and a unit grid spacing of 0.375 Å. The center of the grid box was located at 136.844, 115.391, and 42.313. For Aβ_1−42_, the crystal structure of Aβ_1−42_ (PDB ID: 2BEG)^63^ was used. The grid map was set up with a grid box size of 74 × 72 × 30 and a unit grid spacing of 0.375 Å. The center of the grid box was located at -8.777, 1.119, 0.253.

For docking calculation, Gasteiger charges were added. The Lamarckian genetic algorithm was selected for molecular docking with a population size of 150 and a ligand orientation of 150. The docking process was set to a maximum of 1,000,000 evaluations and 27,000 generations. Binding affinity was assessed based on the calculated binding energy (kcal/mol). Furthermore, the protein-compound complex was visualized using BIOVIA Discovery Studio Visualizer 2025.

#### Molecular Dynamics (MD) Simulations

The MD simulations were performed on three systems: the apo BACE1 structure, the BACE1-ligand complex, and the BACE1-BACE1 inhibitor IV complex, using GROMACS 2025.4^64^. The AMBER99SB-ILDN force field^65^ was used to define the protein topology, while the General Amber Force Field (GAFF2) force field, generated with the ACPYPE server^66^, was used to define the ligand topologies. For system preparation, the complexes were placed in a dodecahedron box with a 1.2 nm buffer from the box boundary and solvated using the TIP3P model. System charges were neutralized by adding Na + and Cl- counter-ions. Energy minimization was performed using the steepest descent algorithm until the maximum force was less than 1000 kJ/mol/nm. Subsequently, the equilibration at constant volume (NVT) was performed at 298.15 K using a v-rescale thermostat^67^ with a time constant of 1.0 ps. The system was further equilibrated at constant pressure (NPT) to achieve a pressure of 1.0 bar by using the c-rescale barostat^68^ with a time constant of 5.0 ps. Production runs were conducted for 500 ns on the LANTA high-performance computing system (ThaiSC, NSTDA), utilizing AMD EPYC™ 7713 CPUs and 4x Nvidia A100 GPUs. Trajectories were saved every 10 ps for analysis. The stability and flexibility of the protein-ligand complex were evaluated by monitoring the root mean square deviation (RMSD) and the root mean square fluctuation (RMSF), respectively. The compactness of the protein was assessed by using the radius of gyration (RoG) analysis. Additionally, the hydrogen bond analysis was performed to investigate the intermolecular interactions using Visual Molecular Dynamics (VMD)^69^.

### *In vitro* assay for AD pathogenesis-related activities

#### β-secretase inhibition activity assay

The β-secretase 1 inhibitory activity of the potential compounds was investigated using an *in vitro* cell-free fluorescence resonance energy transfer (FRET) assay with a BACE1 activity detection kit. β-secretase inhibitor IV was used as a positive control. Test compounds were dissolved in 100% DMSO (Dimethyl sulfoxide) to prepare a 10 mM stock solution, which was used to prepare final concentrations ranging from 0.1 to 1000 µM for the screening assay and IC_50_ investigation. The reaction was conducted on a 96-well black plate with a total volume of 100 µL. The reaction mixture consisted of 2 µL of test compound solution and 20 µL of 50 µM BACE1 substrate. The volume of the reaction mixture was adjusted to 100 µL by adding fluorescence assay buffer. Before the test, the reaction was initiated by adding 2 µL of 0.3 unit/µL BACE1. The fluorescence intensity was measured kinetically every 1 min for 2 h at 37 °C using a Tecan Infinite^®^ 200 PRO multimode reader (excitation 320 nm, emission 405 nm)^70^.

#### Cholinesterase inhibition activity assay

The inhibitory activities of the potential compounds against AChE and BuChE were evaluated using a modified Ellman’s method. For both assays, Test compounds were dissolved in 100% ethanol to prepare a 10 mM stock solution, which was used to prepare final concentrations ranging from 0.1 to 1000 µM for the screening assay and IC_50_ investigation. Experiments were conducted in 96-well plates with a total volume of 250 µL. This reaction mixture consisted of 25 µL of the test compound, 125 µL of 1 mM DTNB, and 25 µL of 0.1 M phosphate buffer (pH 7.4). To initiate the reaction, 50 µL of either 0.2 U/mL AChE (type VI-S) or 0.2 Units/mL BuChE was added, along with 25 µL of either 1 mM ATCI for AChE or 1 mM BTCI for BuChE as the substrate. Tacrine served as a positive inhibitor in both assays. The absorbance was measured kinetically at 405 nm every 30 s for 5 min at 25 °C using an Ensight™ Multimode reader (PerkinElmer, Massachusetts, United States)^71^.

#### Amyloid β aggregation inhibition assay

The Aβ _1−42_ aggregation inhibition assay of the potential compound was evaluated by using the thioflavin-T (ThT) assay. Test compounds were dissolved in 100% DMSO to prepare a 10 mM stock solution, which was used to prepare final concentrations ranging from 0.1 to 1000 µM for the screening assay and IC_50_ investigation. The reactions were performed in a 96-well black plate by incubating 2 µL of the test compound solution with 18 µL of 10 µM Aβ_1–42_ solution in 0.5 M phosphate buffer (pH 7.4) at 37 °C for 48 h. Before measuring, 180 µL of a 5 µM ThT solution was added, and the mixture was gently mixed with the test solution. The fluorescence intensities were measured using an Ensight Multimode reader (PerkinElmer, Massachusetts, United States), at excitation 446 nm and emission 490 nm. Curcumin was used as a positive inhibitor^72^.

#### ABTS radical scavenging assay

The antioxidant activity of the test compound was evaluated by using the ABTS radical scavenging assay. Test compounds were dissolved in 100% ethanol to prepare a 10 mM stock solution, which was used to prepare final concentrations ranging from 0.1 to 1000 µM for the screening assay and IC_50_ investigation. The oxidation reaction between 2.45 mM K_2_S_2_O_8_ and 7 mM ABTS in deionized water (1:1 v/v) generates ABTS^•+^ radical. The reaction solution was incubated for 12–16 h. The ABTS radical scavenging activity of the test compound was assessed by incubating 50 µL of the test compound solution with 100 µL of ABTS^•+^ solution at room temperature, protected from light, for 15 min at 25 °C. The total volume of the reaction mixture is 150 µM. The absorbance was recorded by using an Ensight™ Multimode reader (PerkinElmer, Massachusetts, United States) at 734 nm. Trolox served as an antioxidant standard.

### Neuroprotective activities assay

The SH-SY5Y cells (ATCC-CRL 2266, A.N.H. Scientific Marketing Co., Ltd., Thailand) were cultured in Dulbecco’s modified Eagle’s medium/nutrient mixture F-12 Ham with 10% FBS, under standard conditions (37 °C, 5% CO₂, humidified atmosphere). Before the experiment, the cells were distributed into 96-well plates at a density of 5 × 10^5^ cells/mL in 100 µL per well and incubated for 48 h. Test compounds were dissolved in 100% DMSO to prepare a 20 mM stock solution, which was used to prepare final concentrations ranging from 0.1 to 100 µM.

#### Cytotoxicity screening

For cytotoxicity screening, cells were treated with the test compound at concentrations of 0.1, 1, 10, and 100 µM for 2 h. Following this incubation, the medium was substituted with 0.5 mg/mL MTT solution, and cells were incubated for an additional 2 h. The resulting formazan crystals were dissolved using a solubilization solution (DMSO), and the absorbance was measured at 550 nm using an Infinite^®^ 200 PRO Multimode Reader (Tecan Group Ltd., Switzerland). The concentration of the potential compound with no cytotoxicity was chosen for further assay.

#### Neuroprotective activities on H_2_O_2_-induced oxidative stress in SH-SY5Y cells

The neuroprotective potential of the compound against oxidative stress was investigated using an MTT colorimetric assay in SH-SY5Y neuroblastoma cells. Hydrogen peroxide (H_2_O_2_) was used to induce oxidative stress cell damage. To determine the test compound’s neuroprotective activity against oxidative damage, cells were pretreated with various concentrations of the test compound for 2 h. The unabsorbed compounds were removed. The H_2_O_2_ solution was exposed to the cell to induce oxidative stress. After 2 h of incubation, cell viability was assessed by MTT colorimetric assay using a Multimode reader Infinite^®^ 200 PRO (Tecan Group Ltd., Switzerland) at 550 nm. NAC was used as a standard^73^.

### Effect of test compound on AD-related protein expression in the SH-SY5Y neuroblastoma cell lines

To investigate the effect of the compound on the expression of an AD-related protein, SH-SY5Y neuroblastoma cells at a density of 1 × 10^6^ cells/mL were seeded into a 6-well plate and incubated for 48 h. The cells were pretreated with a potential compound or a standard compound (NAC) and incubated for 2 h. After 2 h, the unabsorbed compounds were removed, and the oxidation stress condition was induced by incubating with a 250 µM H_2_O_2_ solution for 7 min. After that, the media with H_2_O_2_ was removed. The lysate buffer was added to the cell culture and incubated for 30 min to lyse the cells. The lysate was centrifuged for 10 min at 4 °C at 13,500 rpm. The supernatant was collected. The Bradford test was used to measure the total protein content. For Western blot analysis, the SDS-PAGE method is used to separate and transfer protein into a polyvinylidene difluoride membrane. To identify the protein bands, the membrane was incubated with primary antibodies targeting APP, BACE, p-GSK-3α/β, p-Akt, p-Tau, cleaved caspase-3, and β-actin. The membrane was then incubated with secondary antibodies. The protein bands were captured using ImageQuant LAS 4000 (GE Healthcare Life Sciences, Sweden). The band intensities were measured by using ImageJ^74,75^.

### Statistical analysis

The data were represented as means ± SD (*n* = 3) for *in vitro*. Statistical significance was assessed by one-way analysis of variance (ANOVA). For all statistical analyses, a *p*-value < 0.05 was considered significant.

## Supplementary Information

Below is the link to the electronic supplementary material.

Supplementary Material 1

## Data Availability

The data generated in the present study may be requested from the corresponding author.
