# Supplementary material for: Repurposing FDA-approved drugs as multi-target neuroprotective agents for Alzheimer’s disease via computational screening and experimental validation
Source: Sci Rep. 2026 Apr 7;16:11688. doi: 10.1038/s41598-026-46708-2 (PMC13062044; doi:10.1038/s41598-026-46708-2)
Supplement: Supplementary file 1 — Supplementary Material 1 [file 41598_2026_46708_MOESM1_ESM.pdf]

Supplementary Materials

Repurposing FDA-Approved Drugs as Multi-Target Neuroprotective Agents for Alzheimer's Disease via Computational Screening and Experimental Validation

Khemjira Phemphunananchai [1], Pornthip Waiwut [2], Jutarop Phetcharaburanin [3], Pattaporn Poonsawas [1], Chantana Boonyarat [1,*]

[1]*Faculty of Pharmaceutical Sciences, Khon Kaen University, 40002, Khon Kaen, Thailand; khemjira_ph@kkumail.com (K.P.), pattpoon@kku.ac.th (P.P.), chaboo@kku.ac.th (C.B.)*

[2]*Faculty of Pharmaceutical Sciences, Ubon Ratchathani University, Ubon Ratchathani 34190, Thailand; porntip.w@ubu.ac.th (P.W.)*

[3]*Department of Systems Biosciences and Computational Medicine, Faculty of Medicine, Khon Kaen University, Khon Kaen, Thailand; jutarop@kku.ac.th (J.P.)*

** Correspondence: Dr.Chantana Boonyarat, Faculty of Pharmaceutical Sciences, Khon Kean University, 123 Village No. 16, Mittraphap Road, Nai-Muang, Muang District, Khon Kean 40002, Thailand; E-mail: chaboo@kku.ac.th; Tel.: +66-81-3073313, +66-43-202305*

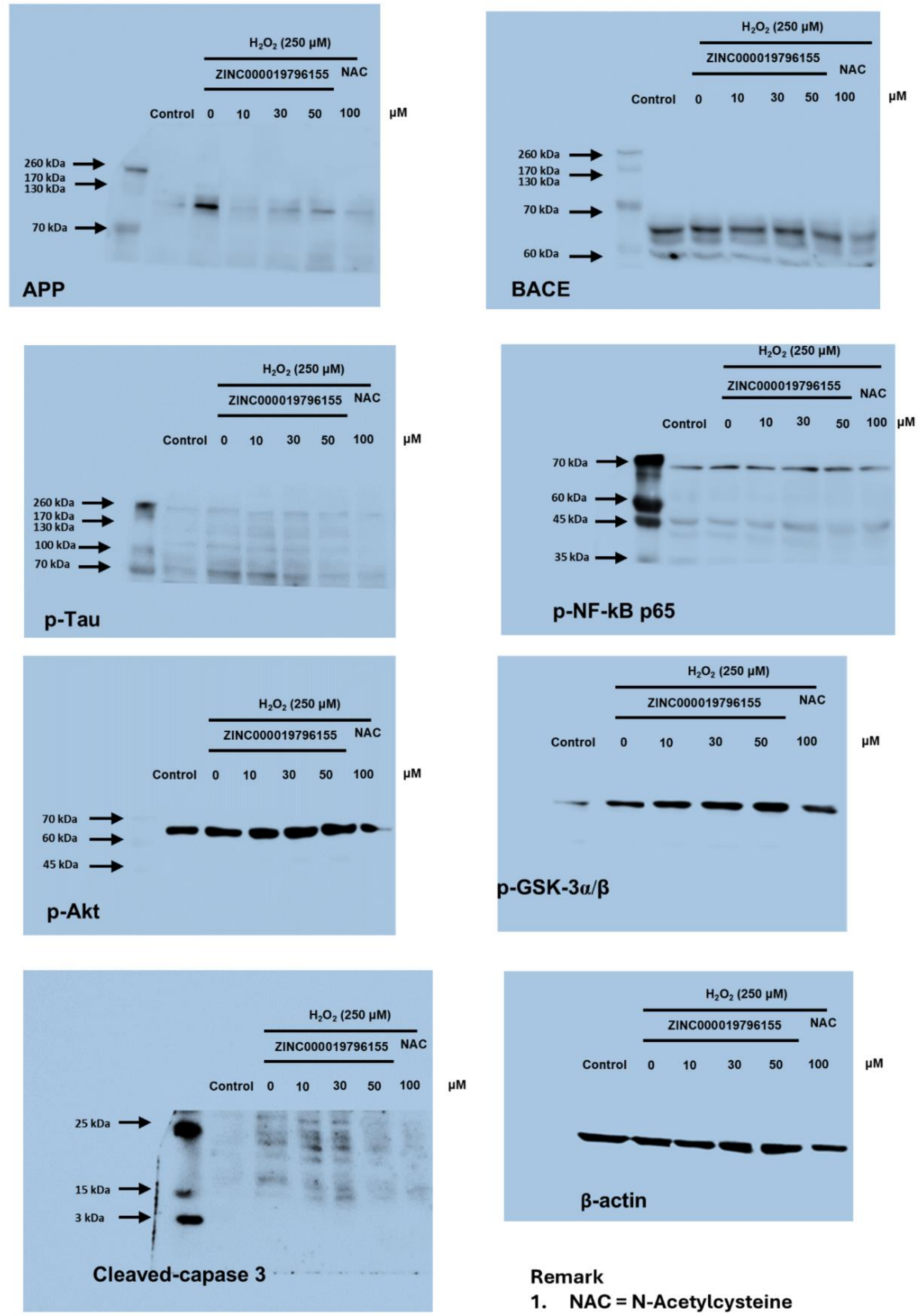

Figure S1. Original blots for Cropped western blot Data in Figure 4a
Original blots for cropped western blot data of APP, BACE, p-Tau, p-NF-κB p65, p-Akt, p-GSK-3-α/β, cleaved-caspase 3, and β-actin in Figure 4a

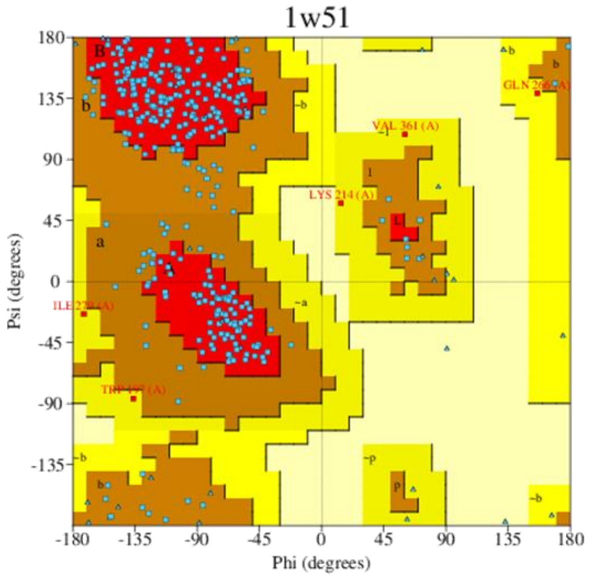

1. Ramachandran Plot statistics

```
                                                  No. of
                                                 residues     %-tage
                                                  ------      ------
Most favoured regions       [A,B,L]                  245       76.8%**
Additional allowed regions  [a,b,l,p]                 69       21.6%
Generously allowed regions  [~a,~b,~l,~p]              5        1.6%
Disallowed regions          [XX]                       0        0.0%
                                                    ----      ------
Non-glycine and non-proline residues                 319      100.0%

End-residues (excl. Gly and Pro)                       6

Glycine residues                                      32
Proline residues                                      18
                                                    ----
Total number of residues                             375
```

Figure S2. The Ramachandran Plot and statistics of the BACE1 (PDB ID: 1w51)

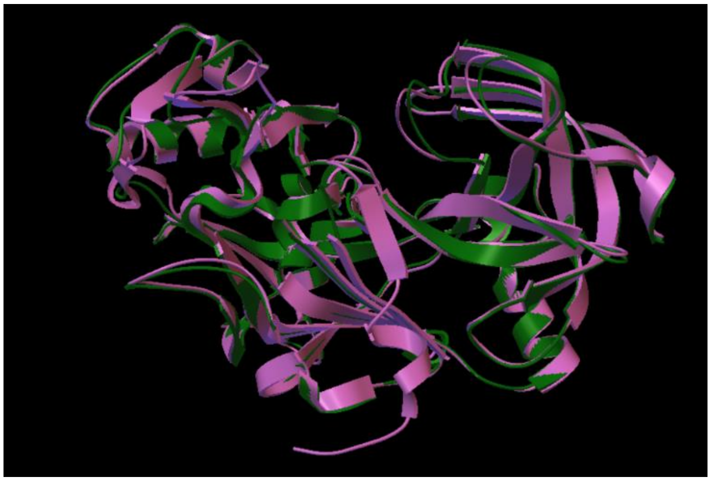

Figure S3. Superimposition of the target protein BACE-1 (PDB: 1W51, shown in green) against the high-resolution reference structure (PDB: 2B8L, resolution 1.70 Å, shown in pink). The Calculated RMSD is 1.027 Å.

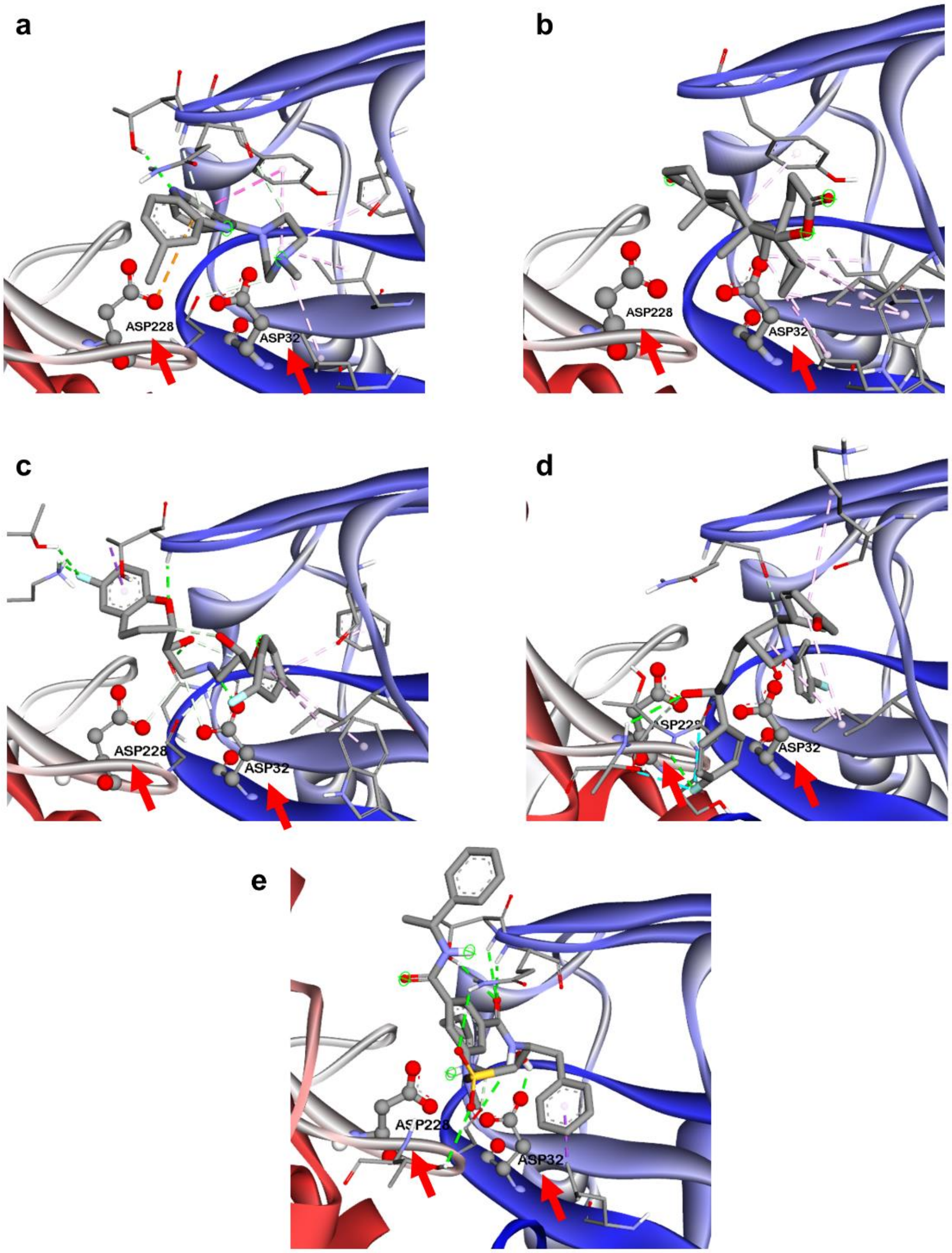

Figure S4. The 3D binding pose of top ligands and reference standards against BACE1's active site. The ligands include ZINC000019796155 (a), ZINC000003927200 (b), ZINC000005844788 (c), ZINC000003810860 (d), respectively. BACE1 inhibitor IV is the reference standard (e). The figure showed the binding positioning over the catalytic aspartic acids, Asp32 and Asp228 (shown in ball and stick form), and binding interaction with the crucial residues in BACE1's active site. The red arrow indicated the catalytic dyad.

Table S1. Sequential filtering of top docking hits based on Lipinski's Rule of Five, Oral Dosage form availability, and geriatric safety profiles.

Zinc number	Docking score (kcal/mol)	MW (<500)	Rotatable bonds (<10)	H-bond acceptors (<10)	H-bond donors (<5)	LOGP (<5)	TPSA (<140)	Clinical indication	Oral Dosage form availability (Yes/No)	Chronic Oral Use Safety?	Selection Decision	Reason for Exclusion
**ZINC000253630390**	-9.9	875.1	8	14	3	4.1	170	Parasite infection	Yes	N/A	Excluded	- Violates Lipinski's Rule of Five<br>- Lack of chronic use studies.
**ZINC000003927200**	-9.7	366.49	0	3	0	2.47	43.37	Oral contraceptives	Yes	Yes	Selected	
**ZINC000000537928**	-9.4	477.04	8	3	1	5.01	43.78	Diarrheal	Yes	No	Excluded	- Violates Lipinski's Rule of Five<br>- Central Nervous System (CNS) side effects
**ZINC000036701290**	-9.4	532.56	6	8	1	4.11	65.77	Chronic Myeloid Leukemia	Yes	No	Excluded	- Anti-neoplastic (cancer) agents<br>- Cardiovascular toxicity
**ZINC000052716421**	-9.4	390.4	5	5	1	3.26	44.27	Hypoactive sexual desire disorder	Yes	Yes	Excluded	- Contraindicated in male patients
**ZINC000005844788**	-9.2	405.44	6	7	3	4.04	70.95	Hypertension, chronic heart failure	Yes	Yes	Selected	
**ZINC000001542199**	-9.1	351.46	3	3	0	4.9	64.49	Plaque psoriasis, Acne vulgaris	No (Topical/cutaneous)	N/A	Excluded	- No oral alternative available<br>- Lack of chronic use studies.
**ZINC000000601229**	-9	381.9	3	3	0	4.37	38.13	Allergy symptoms	No (Nasal)	N/A	Excluded	- No oral alternative available<br>- Lack of chronic use studies.
**ZINC000000538658**	-8.9	448.94	5	3	2	4.77	69.64	Hyponatremia	Yes	No	Excluded	- Severe liver injury risk: May require transplantation or cause death
**ZINC000003797541**	-8.9	349.51	1	2	1	4.63	33.12	Prostate cancer	Yes	No	Excluded	- Anti-neoplastic (cancer) agents
**ZINC000003810860**	-8.8	409.43	6	5	2	3.96	60.77	Hypercholesterolemia	Yes	Yes	Selected	
**ZINC000000004893**	-8.7	285.77	0	2	0	3.77	12.47	Schizophrenia, Bipolar	No (sublingual/Transdermal)	Yes	Excluded	- No oral alternative available
**ZINC000019796155**	-8.7	326.82	1	2	1	3.23	30.87	Schizophrenia, Suicidal behavior	Yes	Yes	Selected	
**ZINC000100015048**	-8.7	416.64	6	3	3	5.1	60.69	Hypocalcemia	Yes	Yes	Excluded	- Violates Lipinski's Rule of Five

N/A: Not Available

Table S2. The predicted surface volume of top ligands and tacrine, the reference standard for AChE and BuChE.

Compound	Surface Volume (Å^3)
ZINC000019796155	292.71
ZINC000003927200	364.78
ZINC000005844788	361.96
ZINC000003810860	371.20
Tacrine	187.86
